# *BRCA1 *and *BRCA2 *mutation predictions using the BOADICEA and BRCAPRO models and penetrance estimation in high-risk French-Canadian families

**DOI:** 10.1186/bcr1365

**Published:** 2005-12-12

**Authors:** Antonis C Antoniou, Francine Durocher, Paula Smith, Jacques Simard, INHERIT BRCAs program members, Douglas F Easton

**Affiliations:** 1 Cancer Research UK Genetic Epidemiology Unit, Strangeways Research Laboratory, Department of Public Health and Primary Care, University of Cambridge, Cambridge, UK; 2 Cancer Genomics Laboratory, Oncology and Molecular Endocrinology Research Center, Centre Hospitalier, Universitaire de Québec and Laval University, Québec, Canada; 3 Other members of the INHERIT (INterdisciplinary HEalth Research International Team on BReast CAncer susceptibility) BRCAs involved in clinical aspects of the program are listed in the Acknowledgments section

## Abstract

**Introduction:**

Several genetic risk models for breast and ovarian cancer have been developed, but their applicability to specific populations has not been evaluated. We used data from French-Canadian families to evaluate the mutation predictions given by the BRCAPRO and BOADICEA models. We also used this data set to estimate the age-specific risks for breast and ovarian cancer in *BRCA1 *and *BRCA2 *mutation carriers.

**Methods:**

A total of 195 families with multiple affected individuals with breast or ovarian cancer were recruited through the INHERIT (INterdisciplinary HEalth Research International Team on BReast CAncer susceptibility) BRCAs research program. Observed *BRCA1 *and *BRCA2 *mutation status was compared with predicted carrier probabilities under the BOADICEA and BRCAPRO models. The models were assessed using Brier scores, attributes diagrams and receiver operating characteristic curves. Log relative risks for breast and ovarian cancer in mutation carriers versus population risks were estimated by maximum likelihood, using a modified segregation analysis implemented in the computer program MENDEL. Twenty-five families were eligible for inclusion in the *BRCA1 *penetrance analysis and 27 families were eligible for the *BRCA2 *penetrance analysis.

**Results:**

The BOADICEA model predicted accurately the number of *BRCA1 *and *BRCA2 *mutations for the various groups of families, and was found to discriminate well at the individual level between carriers and noncarriers. BRCAPRO over-predicted the number of mutations in almost all groups of families, in particular the number of *BRCA1 *mutations. It significantly overestimated the carrier frequency for high predicted probabilities. However, it discriminated well between carriers and noncarriers. Receiver operating characteristic (ROC) curves indicate similar sensitivity and specificity for BRCAPRO and BOADICEA. The estimated risks for breast and ovarian cancer in *BRCA1 *and *BRCA2 *mutation carriers were consistent with previously published estimates.

**Conclusion:**

The BOADICEA model predicts accurately the carrier probabilities in French-Canadian families and may be used for counselling in this population. None of the penetrance estimates was significantly different from previous estimates, suggesting that previous estimates may be appropriate for counselling in this population.

## Introduction

*BRCA1 *and *BRCA2 *are the most important breast cancer susceptibility genes identified to date. A recent meta-analysis of 22 population-based studies of breast and ovarian cancer [1] estimated the risk for breast cancer by age 70 years in *BRCA1 *and *BRCA2 *carriers to be 65% and 45%, respectively. The corresponding ovarian cancer risks were 39% and 9%, respectively. A number of other studies have investigated the penetrance of these mutations, and risks have been found to vary by birth cohort, mutation position in the gene, ascertainment criteria and population studied [1-13]. Having precise estimates of these risks is important for counselling mutation carriers, and such estimates are fundamental components of cancer risk prediction models. Such models are currently being used and developed for counselling high risk women in clinical genetics centres [14-17].

Population isolates or founder populations provide a particular challenge for risk models, because the frequency of mutations may be altered by the occurrence of specific founder mutations that may attain relatively high frequency. Notable examples of this are the founder *BRCA1 *and *BRCA2 *mutations in the Ashkenazi Jewish population, and the founder *BRCA2 *population in Iceland [13,18-20]. The French-Canadian population is an example of such a founder population. The province of Quebec population includes about 6 million French-Canadians, who are descendants of about 10,000 immigrants, mostly from France, who settled in Nouvelle France between 1608 and 1760. Altogether, approximately 80% of these founders still have descendants in Quebec today, and they account for the major part of the contemporary French-Canadian gene pool [21,22]. Founder mutations in both *BRCA1 *and *BRCA2 *genes have been characterized in French-Canadian high-risk breast/ovarian cancer families [23-26].

A number of models that predict *BRCA1 *and *BRCA2 *mutation carrier risks and/or breast and ovarian cancer risks have been reported in the literature [14-16,27-29]. The BRCAPRO model was developed using published data and assumes that genetic susceptibility to breast and ovarian cancer is due to mutations in *BRCA1 *and *BRCA2*. The program uses information on first-degree and second-degree relatives to compute the probability that an individual carries a mutation in these genes [16,30]. The recently published BOADICEA (Breast and Ovarian Analysis of Disease Incidence and Carrier Estimation Algorithm) model was developed using a combination of population-based families and families with multiple affected individuals, mainly from the UK. It takes into account the simultaneous effects of *BRCA1*, *BRCA2 *and the residual familial clustering of breast cancer not accounted by these genes, which is assumed to be explained by a polygenic model [14]. The model can be used to compute age-specific cancer risks and mutation carrier probabilities using information on breast and ovarian cancer occurrence in families of any size or structure.

In the present study we used data from families of French-Canadian origin identified through the INHERIT (INterdisciplinary HEalth Research International Team on BReast CAncer susceptibility) BRCAs integrated clinical research program to assess the BRCAPRO and BOADICEA models of genetic susceptibility to breast cancer [14,16] and to investigate whether these models are suitable for counselling women in this population. We also used this data set to estimate the risks for breast and ovarian cancer conferred by *BRCA1 *and *BRCA2 *mutations.

## Materials and methods

### Ascertainment of families

High-risk French-Canadian breast and/or ovarian families were recruited into a research project started in 1996, which subsequently evolved into a large ongoing interdisciplinary research program designated INHERIT BRCAs [31,32]. A major component was to identify and characterize *BRCA1 *and *BRCA2 *mutations in the French-Canadian population. This integrated clinical research program was composed of a network of referring clinicians across the province of Quebec.

Following pre-test education sessions and detailed analysis of familial history, individuals were recruited if their family met one or more of the following strict criteria: the family had at least four individuals with breast and/or ovarian cancer diagnosed at any age in first-degree or second-degree relatives; the family had three first-degree relatives affected with breast and/or ovarian cancer at any age; or the family carried a deleterious mutation already identified in the *BRCA1 *or *BRCA2 *genes. Eight additional families that did not meet those strict criteria were recruited when the analysis of pedigrees was suggestive of a genetic component (e.g. monozygotic twins affected with breast cancer at an early age; four related individuals with early-onset breast cancer; one case of male breast cancer plus a woman affected with early breast cancer). All participants had to be at least 18 years of age and mentally competent. In most instances, the diagnoses of breast and/or ovarian cancer were confirmed by examining a pathology report. Clinicians involved in the research program were responsible for disclosure of the *BRCA1*/*BRCA2 *test result to participants. Approval was obtained from eight ethics committees corresponding to the various institutions participating in the research program.

A total of 191 families were recruited before 1 January 2003, and data from these families were used to assess the ability of the models to predict *BRCA1 *and *BRCA2 *carrier probabilities. Four additional families ascertained by July 2003 were found to segregate *BRCA1 *and *BRCA2 *truncating mutations and were included in the analyses to estimate penetrance, in order to improve the precision of the penetrance estimates.

### Mutation testing

Individuals were assumed to be *BRCA1 *or *BRCA2 *positive only if they carried a clearly deleterious mutation. Carriers of variants of unspecified significance (VUS) were assumed to be *BRCA1*/*BRCA2 *negative for the purpose of these analyses.

Once a signed informed consent form had been obtained from each participant, 40 ml blood was drawn and genomic DNA extracted using the guanidine hydrochloride-proteinase k method [33] and the QIAmp Maxi blood kit (QIAGEN, Mississauga, Canada), in accordance with the manufacturer's instructions. Participants from families considered in the present study were first tested for a panel of 18 truncating mutations detected or reported in the French-Canadian population. This screening led to the discovery of mutations in 52 families (seven distinct mutations), which accounted for 88% of all positive families identified before July 2003. DNA samples from at least one individual from 113 of the families with inconclusive *BRCA1*/*BRCA2 *test results (i.e. no mutation found in the first stage) were sent to Myriad Genetics Laboratories (Salt Lake City, UT, USA) for full-length *BRCA1*/*BRCA2 *sequencing using their Comprehensive BRACAnalysis^®^. No additional analysis was performed for 30 families. Testing services were performed according to the Memorandum of Understanding of the US National Cancer Institute (NCI) for NCI-funded research testing services for *BRCA1 *and *BRCA2 *(project no. NCI 173). This second step screening approach led to the discovery of mutations in seven different families (seven distinct mutations), leaving 106 families with an inconclusive result [24]. DNA samples from 98 families with an inconclusive result were tested at the Cancer Genomics Laboratory at Québec City by multiplex ligation probe amplification. These approaches failed to detect any deleterious rearrangement (Moisan AM, Fortin J, Dumont M, Samson C, Bessette P, Chiquette J, Laframboise R, Lépine J, Lespérance B, Pichette R, Plante M, Provencher L, Voyer P, Goldgar D, Bridge P, Simard J. No evidence of BRCA1/2 genomic rearrangements in high risk French-Canadian breast/ovarian cancer families, submitted, 2005). A confirmation test was performed for each individual belonging to a *BRCA1*/*BRCA2 *positive family on a second blood sample by the Molecular Diagnostic Laboratory of Alberta Children's Hospital (Calgary, Alberta, Canada), under the responsibility of Dr Peter Bridge. VUS were detected in 13 families without deleterious mutations.

### Statistical methods

#### Penetrance estimation

We used data from the families in which deleterious *BRCA1 *or *BRCA2 *mutations were identified to estimate the breast and ovarian cancer risks in carriers of such mutations. For these calculations we considered only those families in which at least one mutation carrier was identified and at least one further family member had DNA testing after the mutation carrier was identified.* BRCA1 *and *BRCA2 *families in which no member was tested for mutations subsequent to the first identified carrier were excluded (seven families). Twenty-five families segregating *BRCA1 *mutations and 27 families segregating *BRCA2 *mutations were eligible for inclusion in the analysis. We used information on the mutation status and disease occurrence in the family members to estimate the breast and ovarian cancer incidence rates in mutation carriers. We used the information available before 2003, with the exception of information about four eligible families segregating a deleterious *BRCA1*/*BRCA2 *mutation, which were recruited by July 2003.

The parameters were estimated by maximum likelihood using a modified segregation analysis implemented in the computer program MENDEL [34]. We assumed a model in which a female after birth was at risk for developing breast or ovarian cancer, or was censored at death or the age at which she was last observed. The disease incidence rates were assumed to depend on the underlying genotype through a Cox proportional hazards model λ_i_(t) = λ_0_(t)RH(t), where λ_0_(t) is the baseline incidence rate for noncarriers and RH(t) is the relative hazard at age t for carriers compared with noncarriers.

Nonmutation carriers were assumed to be susceptible to the population incidence rates for Quebec during 1978–1981 (International Agency for Research on Cancer, Cancer Incidence in Five Continents, Volume V, IARC Scientific publications, Lyon, 1987). The models were parameterized in terms of the age-specific log relative hazard for breast and ovarian cancer compared with the population risks. More details on this method can be found in the report by Antoniou and coworkers [1]. We also performed analyses in which the end-point of interest was either breast or ovarian cancer. A single set of incidence rates was derived representing the incidence of either breast or ovarian cancer in *BRCA1 *or *BRCA2 *mutation carriers. All analyses were performed separately for the *BRCA1 *and *BRCA2 *positive families.

To correct for ascertainment, we employed the sequential ascertainment correction scheme described by Cannings and Thompson [35]. Because family ascertainment was through multiple affected individuals with at least one family member testing positive for mutations, we maximized the conditional likelihood of all phenotypic and genotypic information in the family given all disease phenotypes and the genotypic information up to the point at which the first mutation carrier was identified.

#### *BRCA1 *and *BRCA2 *carrier probabilities

To assess the consistency of the Quebec families with genetic models that incorporate the effects of *BRCA1 *and *BRCA2 *mutations, we used the data to estimate the *BRCA1 *and *BRCA2 *mutation carrier probabilities of the first screened individual in each family. For this analysis we used data from the 191 families recruited between 1996 and 2003, and considered only those families in which mutation screening was carried out in at least one family member. The families of individuals who were recruited because of prior knowledge of a mutation segregating within the family were excluded (three in total), thus leaving a total of 188 families. For the purpose of these predictions, the first individual in whom mutation screening was carried out was considered the proband, and predictions were only made for these individuals. In some instances a mutation was identified after screening several individuals in the family, even though the first tested individual was found to be a noncarrier. These subsequent rounds of testing were ignored, because the predicted probabilities for subsequent individuals would be altered by the fact that a mutation had not been found previously. We used the information on breast and ovarian cancer diagnosis in families that was available before 2003.

#### Mutation prediction models

BOADICEA is a computerized risk assessment program that can be used to compute the probability of detecting a *BRCA1 *or *BRCA2 *mutation and the risks for developing breast or ovarian cancer [14]. The model considers the occurrence of breast and ovarian cancer within the family. In the current version, only the first cancer in an individual is considered. Disease occurrence in males and cancers other than breast and ovarian cancers are ignored. The model is a genetic model that is implemented in the program MENDEL [34], so that families of any size or structure can be included. In the model, *BRCA1 *mutations are assumed to have a population allele frequency of 0.06% and *BRCA2 *mutations to have an allele frequency of 0.10%. The residual familial clustering not accounted for by *BRCA1 *and *BRCA2 *mutations is assumed to be explained by a polygenic model with a variance that decreases with increasing age [14]. We used an updated version of the model in which the cancer risks in both carriers and noncarriers also depend on the year of birth of the individual (Antoniou and coworkers, unpublished data). For example, the risk for breast cancer by age 70 years is 46% for a *BRCA1 *carrier born before 1920, but it is 59% if the carrier is born after 1950. For these analyses, the sensitivity of mutation detection was assumed to be 70% for *BRCA1 *and 80% for *BRCA2 *(Easton D, based on Breast Cancer Linkage Consortium data; personal communication).

The BRCAPRO program computes the probability that an individual carries a *BRCA1 *or a *BRCA2 *mutation based on information provided on relatives as distant as second degree [16,36]. The model assumes that genetic susceptibility to breast cancer is entirely due to mutations in *BRCA1 *and *BRCA2*. We used the version implemented in the BayesMendel software, which is a library of programs written in the R programming environment [37,38]. BRCAPRO, as distributed in BayesMendel, comes with various options for *BRCA1 *and *BRCA2 *penetrance and allele frequencies. For our purposes we assumed the non-Ashkenazi allele frequencies (*BRCA1*: 0.0006; and *BRCA2*: 0.00022). We also used the current default penetrances of BRCAPRO for populations other than Ashkenazi Jewish.

To provide a more direct comparison between the models, two sets of predictions were carried out under the BOADICEA model: taking into account the entire family as reported, which we refer to as 'full pedigrees'; and taking into account only the first-degree and second-degree relatives of the first screened individual.

### Model comparisons

The models were calibrated by comparing the observed and expected numbers of mutations. The models were also evaluated in terms of the Brier score (BS) [39]. This is a measure of accuracy of the predictions that measures the total difference between observing a mutation and the predicted probability of detecting a mutation. This is defined as follows:



Where *n *is number of families, *p*_*i *_is the probability of detecting a *BRCA1 *or *BRCA2 *mutation, and *x*_*i *_is 0 if the proband was found not to carry a mutation and 1 if the proband was found to carry a *BRCA1 *or *BRCA2 *mutation. The smaller the BS, the closer are the predictions to the observed data. The significance of the score was evaluated by the means of the Spiegelhalter z-statistic [40].

The models were also evaluated in terms of their ability to predict accurate probabilities (reliability), and the ability of the predictions to separate correctly the carriers and noncarriers (resolution/discrimination). These were assessed in terms of the attributes diagrams. This involved ranking the predicted probabilities and then dividing them into quantiles. The average predicted probability was computed for each quantile and this was then plotted against the observed mutation frequency in the quantile. Ninety-five per cent confidence intervals (CIs) for the observed frequencies were computed using a binomial distribution.

Receiver operating characteristic (ROC) curves were used to assess the specificity and sensitivity of the various models. Sensitivity here is defined as the proportion of tested individuals with mutations with a detection or carrier probability higher than a given value (cutoff), and specificity is the proportion of individuals without mutations with a detection probability lower than the cutoff. The ROC curves plot the sensitivity against the specificity at all possible cutoffs and show the trade-off between the two measures. The area under the ROC curve is a measure of the accuracy of a model, such that the higher the area, the more accurate is the model (an area of 1 represents a perfect fit, and an area of 0.5 represents no predictive value).

## Results

### *BRCA1 *and *BRCA2 *mutation prediction

A total of 191 families were recruited before January 2003. Three of those families were recruited because of prior knowledge of a mutation segregating within the family and were therefore excluded from this analysis. Table 1 shows some summary statistics for these families. Ninety one percent of families were extended beyond second-degree relatives. In 104 families the first individual screened for mutations was unaffected, in 73 families the first screened individual had developed breast cancer, and in the remaining 11 families the first screened individual had developed ovarian cancer. Table 2 shows the predicted number of mutations in those individuals under the BOADICEA and BRCAPRO models. As described in the Materials and methods section (above), BRCAPRO considers only second-degree relatives whereas BOADICEA allows for families of an arbitrary size and structure.

Among all the first screened individuals, 14 *BRCA1 *mutations and 19 *BRCA2 *mutations were identified. When information on all reported relatives in each family was considered, the total numbers of mutations predicted by BOADICEA were close to the observed numbers: 15.69 for *BRCA1 *and 20.10 for *BRCA2*. However, the expected number of *BRCA1 *mutations was somewhat lower than observed when only the second-degree relatives were considered (12.15) and much lower for *BRCA2 *(13.26 predicted versus 19 observed). BRCAPRO over-predicted the total number of *BRCA1 *mutations (42.16) and under-predicted the number of *BRCA2 *mutations (15.67). The Pearson χ^2 ^goodness-of-fit test for comparing the total number of observed mutations with the predicted number was not significant for BOADICEA but was highly significant for BRCAPRO (*P *= 6 × 10^-6^). When information on all available relatives was used, the predicted numbers under BOADICEA were close to the observed numbers for both unaffected probands and among the breast and ovarian cancer patients. BRCAPRO over-predicted the number of *BRCA1 *mutations among both unaffected individuals and breast cancer cases.

Table 3 shows the predicted number of mutations by the number of breast and ovarian cancer cases in the family diagnosed under age 70 years. The number of *BRCA1 *and *BRCA2 *mutations predicted by BOADICEA when the 'full pedigrees' were considered was close to the observed number in each subcategory except among families with four or more breast cancer cases, for which the number of predicted *BRCA1 *mutation was higher than the number observed (6.20 versus 2). When information on family members was restricted to the second-degree relatives of the first screened individual, the under-prediction of *BRCA1 *mutations was mainly among families with at least three cases of breast cancer and at least one case of ovarian cancer (3.12 expected versus 6 observed). The expected number of *BRCA1 *mutations was also somewhat higher than the observed number among families with at least four cases of breast cancer cases and no ovarian cancer cases (4.11 versus 2). The under-prediction of *BRCA2 *mutations was mainly within this category of families (8.60 expected versus 12 observed). BRCAPRO over-predicted the number of *BRCA1 *mutations in all categories, but especially among families with at least four breast cancer cases and no ovarian cancer cases (19.40 versus 2). Within the same family category, the number of *BRCA2 *mutations was under-predicted (8.27 expected versus 12 observed).

The BS for the probability of detecting a *BRCA1 *or a *BRCA2 *mutation under the BOADICEA model was 0.112 when data from the 'full pedigrees' were used to carry out the predictions and 0.118 when information was restricted to second-degree relatives only. The null hypothesis that the BOADICEA predictions are compatible with the observations was not rejected when the full pedigrees were used but was rejected when family history information was restricted to second-degree relatives only (*P *= 0.61 and *P *= 0.02, respectively, using Spiegelhalter's z-statistic). Under BRCAPRO the BS was 0.148 (*P *= 0.003).

### Resolution and reliability

**Figure 1:**
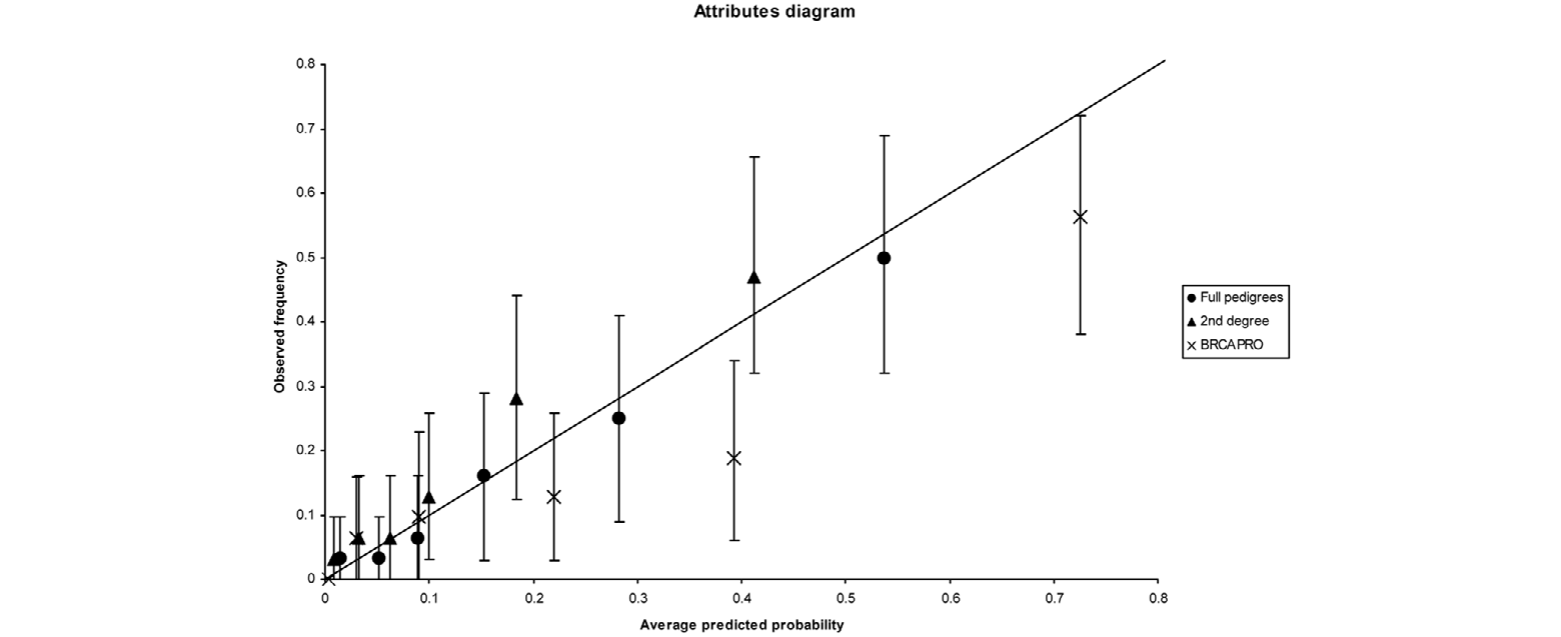
Attributes diagram. Shown is an attributes diagram comparing the predicted carrier probabilities and the observed carrier frequencies for *BRCA1 *and *BRCA2 *mutations combined. BOADICEA predictions were carried out using data from all reported family members and restricted to second-degree relatives only. BRCAPRO predictions were adjusted for an 80% sensitivity for the mutation screening methods used. Each point represents a sextile of the data and 95% confidence intervals were computed assuming a binomial distribution.

Figure 1 shows the attributes diagram for evaluating the reliability and resolution of the models. In this case the predicted carrier probabilities were ranked and divided into sextiles so that each quintile contains 31 or 32 observations. For this purpose the BRCAPRO carrier probabilities were multiplied by 0.80 to allow for reduced sensitivity of mutation testing (i.e. assuming that only 80% of the mutations could be identified with the screening methods used). Points on the 45° line indicate both good reliability and resolution, whereas points above the line suggest that a model makes under-confident predictions and in effect provides poor discrimination and inaccurate probabilities. Points below the line suggest good discrimination but inaccurate probabilities.

The average predicted probability in each sextile was very close to the observed frequency under the BOADICEA model when the 'full pedigrees' were considered, indicating that under such circumstances the model discriminates between carriers and noncarriers and it gives accurate probabilities. When only the second-degree relatives were considered the average predicted probability was slightly lower than the observed frequency in each sextile, indicating somewhat lower discrimination. However, the 95% CIs of the observed frequencies for all sextiles include the average predicted probabilities. BRCAPRO provided very good discrimination between carriers and noncarriers, but it did not give accurate probabilities, especially at the two upper sextiles. In these cases the average predicted probabilities were much higher than the observed frequencies and outside their 95% CIs.

**Figure 2:**
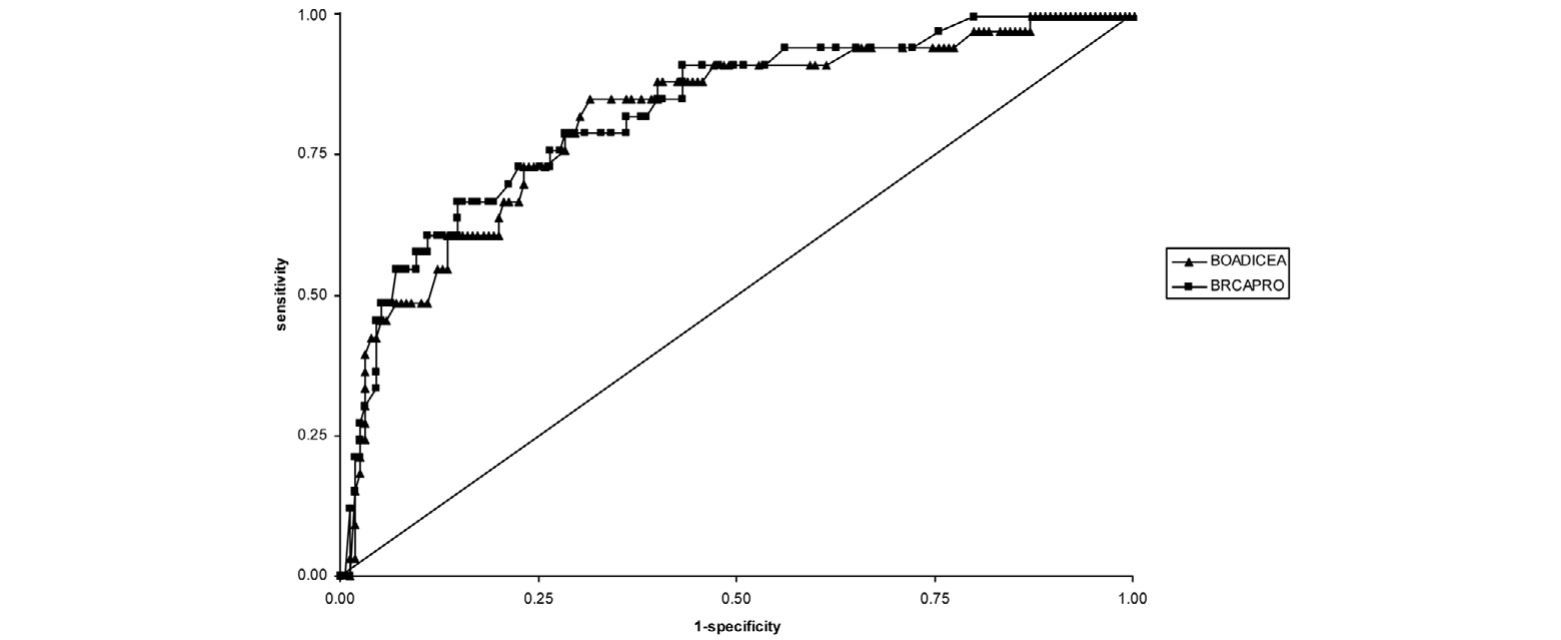
ROC curves. Shown are ROC curves for the BOADICEA and BRCAPRO predictions of carrying either a *BRCA1 *or a *BRCA2 *mutation. BOADICEA predictions assumed all reported family members. ROC, receiver operating characteristic.

The ROC curves under BOADICEA using the full pedigrees and under BRCAPRO are shown in Fig. 2. The area under the curve was 83% (95% CI 75–91%) under BRCAPRO and slightly lower, at 81% (95% CI 73–90%), under BOADICEA. However, the difference was not statistically significant. The curves can be used to define cutoffs for referring individuals for *BRCA1 *and *BRCA2 *mutation screening. Under BOADICEA, if all individuals with mutation detection probability of 16% and over were referred for screening, a sensitivity of approximately 82% would be achieved. To obtain the same sensitivity under BRCAPRO, the mutation carrier probability cut-point needs to be approximately 25%. At these cutoffs the BOADICEA predictions are 69% specific and the BRCAPRO predictions 64% specific. The positive predictive value of BOADICEA was 36% and the negative predictive value 95%. Under BRCAPRO the positive predictive value was 32% and the negative predictive value was 94%. Carriers and noncarriers were correctly classified 72% and 67% of the time under BOADICEA and BRCAPRO, respectively.

### Risks for breast and ovarian cancers

Table 4 shows the estimated cumulative risks for breast and ovarian cancer in *BRCA1 *and *BRCA2 *mutation carriers. The risks were estimated separately for *BRCA1 *and *BRCA2 *positive families. Twenty-five families were used in the *BRCA1 *analysis. The cumulative risk for breast cancer by age 50 years was estimated to be 20% (95% CI 0–45%) and by age 70 years it was estimated to be 72% (95% CI 0–93%). The corresponding ovarian cancer cumulative risks were estimated to be 1% (95% CI 0–10%) and 38% (95% CI 0–78%). When the event of interest was defined to be either breast or ovarian cancer, the cumulative risks for either cancer were estimated to be 23% by age 50 years (95% CI 0–48%) and 83% by age 70 years (95% CI 34–96%). When the analyses were restricted to the 18 families carrying the founder mutation R1443X, the cumulative risk for breast cancer was estimated to be 86% by age 70 years and the cumulative ovarian cancer risk 54%.

Data from 27 families were used in the *BRCA2 *analyses. The cumulative risk for breast cancer was estimated to be 21% by age 50 years (95% CI 0–55%) and 75% by age 70 years (95% CI 0–97%). The corresponding cumulative risks for ovarian cancer were 0.4% (95% CI 0–2%) and 49% (95% CI 0–81%). The cumulative risk for developing either breast or ovarian cancer was estimated to be 35% by age 50 years (95% CI 0–64%) and 89% by age 70 years (95% CI 34–98%). When only the 23 families segregating the *BRCA2 *founder mutation 8765delAG were used, the cumulative breast cancer risk by age 70 was estimated to be 71% and the corresponding ovarian cancer cumulative risk to be 51%.

## Discussion

In the present study we used data from French-Canadian families included in the INHERIT program to evaluate the mutation risk prediction models BOADICEA and BRCAPRO [14,16] and to estimate the breast and ovarian cancer risks conferred by *BRCA1 *and *BRCA2 *mutations. A total of 157 of the families were screened for mutations using a three-step approach: screening for the 18 known French-Canadian mutations, full sequencing, and multiplex ligation probe amplification for detecting large rearrangements. Eight families were screened using only the first two steps and 30 families were only screened for the known French-Canadian mutations. However, the two additional steps detected only seven mutations, and it is therefore likely that no more than one or two mutations were missed.

Penetrance was estimated using data from families segregating *BRCA1 *and *BRCA2 *mutations. The breast cancer risk by age 70 years was estimated to be 72% in *BRCA1 *mutation carriers and 75% in *BRCA2 *mutation carriers. The corresponding ovarian cancer risks were 38% and 49%, respectively. These point estimates are similar to the risks derived in previous studies conducted in families with multiple affected individuals [2,3,41]. However, our estimates are associated with very wide confidence intervals, and the present risks are also not significantly different from those derived using families identified through population-based series of breast or ovarian cancer patients [1,4,6-8,42]. The large confidence intervals are partly due to the relatively small number of mutation-positive families considered in the estimation (25 for *BRCA1 *and 27 for *BRCA2*) but most importantly are due to the strong ascertainment correction. In our analyses we conditioned on the phenotypes of all family members and the genotypes of all individuals up to and including the first identified mutation carrier in the family. Therefore, the information contributing to the analyses only came from individuals typed after the first mutation carrier was identified. The precision of these estimates may be improved by genotyping additional family members or by adding newly identified mutation-positive families in the analysis.

The BOADICEA and BRCAPRO genetic models for breast cancer susceptibility were used to compute expected numbers of mutations within subcategories of families. Empirical models such as the Myriad II model [29] and the Manchester scoring system [43] were used in these comparisons because they will be investigated in a future report (Simard and coworkers, unpublished data). The predicted numbers of mutations under BOADICEA were very close to the observed numbers when information on all reported relatives was taken into account. However, when only the second-degree relatives of the proband were considered the model under-predicted somewhat. This is consistent with the general principle that risk predictions should, where possible, take into account all known information.

The total number of mutations was significantly over-predicted by BRCAPRO. This was due to the over-prediction of *BRCA1 *mutations in almost all categories, with an under-prediction of the number of *BRCA2 *mutations. Even if the predicted number of mutations is adjusted for the reduced sensitivity of the mutation detection techniques used, BRCAPRO would still over-predict the number of *BRCA1 *mutations. The over-prediction of *BRCA1 *mutations does not seem to be due to the mutation frequency or penetrance estimates used in the models. The penetrance estimates used in the current implementation of BRCAPRO were the latest for non-Ashkenazi carriers, which are lower than the Breast Cancer Linkage Consortium estimates used in the initial implementation [2,3] and are closer to the estimates used in BOADICEA, although they are not cohort specific. As implemented here, both BOADICEA and BRCAPRO use a *BRCA1 *mutation frequency of 0.06%. BOADICEA uses a higher *BRCA2 *mutation frequency than does BRCAPRO (0.10% versus 0.02%). However, when the *BRCA2 *frequency in BRCAPRO was assumed to be the same as in BOADICEA, BRCAPRO over-predicts the number of mutations for both *BRCA1 *and *BRCA2 *(predicted numbers: 29.42 and 38.18, respectively). Presumably, this discrepancy results from the fact that BRCAPRO does not model any of the residual familial clustering of breast cancer, other than *BRCA1 *or *BRCA2*, so that individuals with moderate family histories are assigned probabilities for being mutation carriers that are too high.

The Brier scores also indicate that BRCAPRO is not accurate in predicting individual carrier probabilities, whereas the BOADICEA predictions are compatible with the observations. On the other hand the ROC curves indicate that BRCAPRO and BOADICEA both discriminate well between carriers and noncarriers. Thus, BRCAPRO and BOADICEA perform similarly in terms of ranking individuals by carrier probability, but the absolute carrier probabilities are only reliable for BOADICEA. To achieve comparable sensitivity and specificity, the cutoffs under the two models are therefore quite different. Users of the models must be aware of these issues when deciding whether to refer an individual for testing on the basis of carrier or mutation detection probabilities given by a particular model.

This is the first time the updated version of BOADICEA has been used to evaluate its prediction of mutation status in an independent data set. The version used here varies from the previous reported model [14] in that the model had been refitted using data from two additional population based studies [44,45] and using a much larger number of *BRCA1 *and *BRCA2 *mutation-positive families [1]. In this respect the penetrance estimates in the latest version are more accurate. Other differences include the use of annual incidence rates as opposed to 5-year interval rates for both carriers and noncarriers, the use of rates that are cohort specific, and incorporation of a polygenic component with a variance that decreases linearly with age. The familial relative risks predicted by this model are closer to the observed risks than those predicted by the previous version (Antoniou and coworkers, unpublished data).* BRCA2 *mutations (and, to a lesser extent, *BRCA1 *mutations) are associated with increased risks for male breast cancer, prostate cancer and pancreatic cancer, and several other cancers have been reported to occur at increased frequency in *BRCA1 *or *BRCA2 *carriers [11,46,47]. The risks for other cancers are not taken into account by the current version of BOADICEA. Incorporating such information into account is likely to result in more accurate carrier probabilities and in better discrimination between carriers and noncarriers, and we are currently extending the model to include such information.

Even though the BOADICEA model was developed using data from the UK, it seems to fit the occurrence of breast cancer in French-Canadian high-risk families. As demonstrated earlier, the penetrance estimates derived from these families are not significantly different from those used in BOADICEA. The good fit to these data suggests that the overall *BRCA1 *and *BRCA2 *allele frequencies are comparable to those in the UK population. This is supported by the fact that studies of prevalence of *BRCA1 *and *BRCA2 *mutations in French-Canadian patients with breast and ovarian cancer unselected for family history indicate similar prevalences of mutations as in the UK population and much lower than the prevalence of mutations among Ashkenazi Jewish women [44,45,48-50].

## Conclusion

In the present study we estimated breast and ovarian cancer risks conferred by *BRCA1 *and *BRCA2 *mutations in French-Canadian families. These estimates are in line with previous ones but they are associated with very large confidence intervals. Additional families or further mutation testing in the families will be necessary to obtain more reliable estimates for this population. The results also suggest that the BOADICEA model of genetic susceptibility fits the data well; therefore, in the absence of reliable estimates, the penetrance functions used in this model can be employed for counselling *BRCA1 *and *BRCA2 *mutation carriers in this population. Using information on all available relatives may improve individual mutation predictions as opposed to restricting information to second-degree relatives only.

## Abbreviations

BOADICEA = Breast and Ovarian Analysis of Disease Incidence and Carrier Estimation Algorithm; BS = Brier score; CI = confidence interval; INHERIT = INterdisciplinary Health Research International Team; NCI = National Cancer Institute; ROC = receiver operating characteristic; VUS = variants of unspecified significance.

## Competing interests

The authors declare that they have no competing interests.

## Authors' contributions

ACA was responsible for the analysis and data cleaning, and led the manuscript preparation. FD is a co-principal investigator in INHERIT BRCAs, initiated and coordinated collaborative efforts, was involved in the study design, and participated in the manuscript preparation. PS was responsible for data cleaning. JS was responsible for supervising mutation screening and laboratory work, initiated the study in high-risk French-Canadian families in 1996 (CBCRA grant), obtained further funding to start the international integrated research program INHERIT BRCAs, and was involved in revising the manuscript. DFE is a co-principal investigator of INHERIT BRCAs, and was involved in the development of the analytical design and in manuscript preparation.

## Figures and Tables

**Table 1:** Summary statistics for the 188 families used in predicting the mutation status of the first screened individual

	Full pedigrees	First- and second-degree relatives only
Mean number of individuals per family	27.9	15.9
Number of BC cases^a^	993	612
Number of OC cases	66	54
Number of bilateral BC cases	70	58
Cases with BC and OC	26	12
Number of male BC cases	19	11
Median age at BC diagnosis (years)	51	51
Median age at OC diagnosis (years)	57	58

^a^Corresponds to females only. BC, breast cancer; OC, ovarian cancer.

BC, breast cancer; OC, ovarian cancer.

**Table 2:** Observed and predicted number of mutations in the first screened individual by cancer status

Cancer status	Model	*BRCA1*^a^	*BRCA2*^a^	Noncarriers
Unaffected	Observed	3	5	96
	BOADICEA			
	Full pedigrees	3.49	6.30	94.21
	Second-degree relatives	2.46	4.10	97.44
	BRCAPRO	10.74	4.20	89.06
Breast cancer	Observed	6	12	55
	BOADICEA			
	Full pedigrees	8.68	13.17	51.14
	Second-degree relatives	7.42	8.75	56.83
	BRCAPRO	26.29	10.90	36.97
Ovarian cancer	Observed	5	2	4
	BOADICEA			
	Full pedigrees	3.52	0.63	6.85
	Second-degree relatives	2.27	0.41	8.31
	BRCAPRO	5.12	0.56	5.32
Totals	Observed	14	19	155
	BOADICEA			
	Full pedigrees	15.69	20.10	152.21
	Second-degree relatives	12.15	13.26	162.59
	BRCAPRO	42.16	15.67	130.35

^a^Not mutually exclusive under BRCAPRO.

**Table 3:** Observed and expected number of mutations among the first screened individuals by number of cancers in the family

Number of cancers, age under 70 years	Model	*BRCA1*		*BRCA2*		Noncarriers	
		
		Observed	Expected	Observed	Expected	Observed	Expected
≥3 BC, ≥1 OC	BOADICEA						
	Full pedigrees	9	6.92	4	3.80	21	23.28
	Second-degree relatives	6	3.12	1	1.49	7	9.38
	BRCAPRO	6	7.80	1	2.35	7	3.98
≥4 BC, 0 OC	BOADICEA						
	Full pedigrees	2	6.20	14	14.75	90	85.05
	Second-degree relatives	2	4.11	12	8.60	57	58.29
	BRCAPRO	2	19.40	12	8.27	57	43.37
3 BC, 0 OC	BOADICEA						
	Full pedigrees	0	0.82	1	1.20	25	23.97
	Second-degree relatives	1	1.09	2	1.77	37	37.13
	BRCAPRO	1	5.43	2	2.97	37	31.60
2 BC, any OC	BOADICEA						
	Full pedigrees	3	1.75	0	0.34	19	19.91
	Second-degree relatives	5	3.83	4	1.40	54	57.79
	BRCAPRO	5	9.53	4	2.08	54	51.50

BC, breast cancer; OC, ovarian cancer.

**Table 4:** Estimated cumulative risks of breast and ovarian cancer in *BRCA1 *and *BRCA2 *mutation carriers

Age (years)	*BRCA1*			*BRCA2*		
	
	BC	OC	BC or OC	BC	OC	BC or OC
30	3	0	4	0.3	0	0.4
40	13	0	14	13	0.2	26
50	20 (0–45)	1 (0–10)	23 (0–48)	21 (0–55)	0.4 (0–2)	35 (0–64)
60	71	38	83	33	49	55
70	72 (0–93)	38 (0–78)	83 (34–96)	75 (0–97)	49 (0–81)	89 (34–98)

Shown are percentage cumulative risks (95% confidence interval). BC, breast cancer; OC, ovarian cancer.
